# N-Check: Nervencheck zur Erfassung einer chemotherapieinduzierten peripheren Neuropathie (CIPN) bei nichtheilbarer Krebserkrankung

**DOI:** 10.1007/s00482-024-00828-8

**Published:** 2024-09-23

**Authors:** Annabell Syben, Sascha Weber, Iris Appelmann, Roman Rolke

**Affiliations:** 1 Klinik für Palliativmedizin, Uniklinik der RWTH, Medizinische Fakultät, RWTH Aachen University, Pauwelsstraße 30, 52074 Aachen, Deutschland; 2 Klinik für Psychiatrie, Psychotherapie und Psychosomatik, Medizinische Fakultät, RWTH Aachen University, Aachen, Deutschland

**Keywords:** Chemotherapieinduzierte Polyneuropathie, CIPN, Neuropathischer Schmerz, QST, Palliative Krebstherapie, Chemotherapy-induced polyneuropathy, CIPN, Neuropathic pain, QST, Palliative chemotherapy

## Abstract

Die chemotherapieinduzierte Polyneuropathie (Synonym: chemotherapie-induzierte periphere Neuropathie, CIPN) umfasst ein Symptomspektrum, das von Hypästhesien mit Störung von Gang‑, Stand- und Feinmotorik bis hin zu schmerzhaften Dysästhesien und Allodynien reicht und die Lebensqualität von Betroffenen maßgeblich beeinträchtigt. In der vorliegenden Pilotstudie wurde mittels quantitativer sensorischer Testung (QST) die CIPN als häufige unerwünschte Wirkung von Zytostatika bei Patient:innen mit einer nichtheilbaren Krebserkrankung untersucht. Die QST ist ein standardisiertes Untersuchungsverfahren, das bei Krebspatient:innen bisher jedoch nicht routinemäßig eingesetzt wird. Sie untersucht thermische und mechanische Wahrnehmungs- und Schmerzschwellen zur Erfassung des subjektiv erlebten Schmerzphänotyps. Im Pilotprojekt N‑Check wurde die QST vor und nach potenziell CIPN-induzierender Therapie eingesetzt und die erhobenen Daten in einer Prä-post-Analyse verglichen. Zusätzlich wurden die spezifischen Auswirkungen der CIPN auf die gesundheitsbezogene Lebensqualität der Patient:innen mithilfe des Fragebogens Functional Assessment for Cancer Therapy – General (FACT-G) erfasst. Insgesamt zeigte sich bei den Patient:innen nach Chemotherapie eine signifikante Hitzehypalgesie als Zeichen einer Schädigung kleiner Nervenfasern. Darüber hinaus fanden sich Zeichen einer Verschlechterung der Lebensqualität. Die Machbarkeit der QST bei Patient:innen mit nichtheilbarer Krebserkrankung und neurotoxischer Chemotherapie konnte in der vorliegenden Pilotstudie gezeigt werden.

**Graphic abstract:**

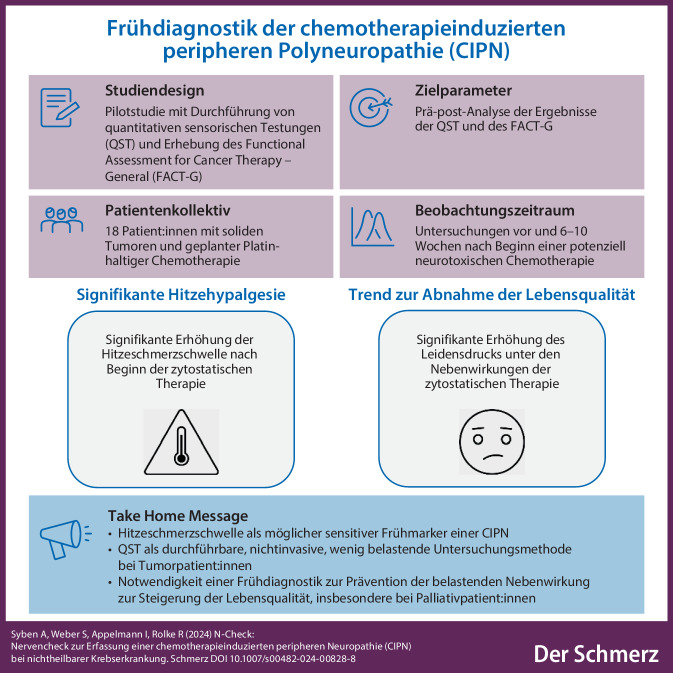

## Abstract (en)

Chemotherapy-induced polyneuropathy (CIPN) encompasses a spectrum of symptoms ranging from hypoesthesia with impaired gait, stance and fine motor skills to painful dysesthesia and allodynia and significantly impairs the quality of life of those affected. In the present pilot study, quantitative sensory testing (QST) was used to investigate CIPN as a common adverse effect of cytostatic drugs in patients with incurable cancer. The QST is a standardized examination procedure that is not yet routinely used in cancer patients. It is used to examine thermal and mechanical perception and pain thresholds to record the subjectively experienced pain phenotype. In the N‑Check pilot project, the QST was used before and after tumor-specific, potentially CIPN-inducing treatment and the data collected was compared in a pre-post analysis. In addition, the specific effects of CIPN on the health-related quality of life of patients treated primarily with a palliative intention were recorded using the Functional Assessment for Cancer Therapy—General (FACT-G) questionnaire. Overall, the patients showed significant heat hypoalgesia after chemotherapy as a sign of damage to small nerve fibers. In addition, there were signs of deterioration of the quality of life. The feasibility of QST in patients with incurable cancer and palliative, neurotoxic chemotherapy was demonstrated in this pilot study.

## Hintergrund und Fragestellung

Die chemotherapieinduzierte periphere Polyneuropathie (CIPN) ist eine sehr häufige Folge tumorspezifischer Therapien [1]. Als auslösende Agenzien sind insbesondere konventionelle Zytostatika, wie Platinderivate, Vincaalkaloide und Taxane, aber auch zielgerichtete Substanzen wie Proteasominhibitoren oder Immuncheckpointinhibitoren zu nennen [1–4].

In einem großen systematischen Review wurde eine substanzunabhängige Prävalenz der CIPN mit 68,1 % im ersten Monat nach Tumortherapie beziffert. Nach drei Monaten lag die Prävalenz noch bei 60,0 % und nach sechs Monaten bei 30,0 % [5].

Die mit den CIPN-bedingten Schmerzen einhergehenden Einbußen an Lebensqualität und Störungen von Gang‑, Stand- und Feinmotorik durch Hyp- und Parästhesien oder extrapyramidal-motorische Funktionsstörungen führen zu einer erheblichen Beeinträchtigung bei den Alltagsaktivitäten bis hin zu einer manifesten körperlichen Behinderung mit entsprechender Hilfs- und Pflegebedürftigkeit [6].

Eine kausale Therapie der CIPN fehlt – abgesehen von einem Entzug der auslösenden Noxe – bislang, ebenso wie eine wirksame medikamentöse oder nichtmedikamentöse Prophylaxe [7, 8]. Auch ist die CIPN nur partiell reversibel [5], und umfassende Verlaufsdaten v. a. auch bei Palliativpatient:innen fehlen bislang. Der Bedarf an wenig invasiver Diagnostik und an verlässlichen Messinstrumenten zur Verlaufsbeurteilung und Prognose bezüglich der allgemeinen Vulnerabilität von Patient:innen ist dementsprechend groß [9]. Daraus ergibt sich die Rationale für die verwendeten Methoden und insgesamt die Notwendigkeit einer routinemäßigen Diagnostik bei CIPN-gefährdeten Patient:innen. Die QST wurde mit der Frage nach ihrer Einsetzbarkeit bei Patient:innen unter CIPN-induzierender Therapie im Rahmen der vorliegenden Pilotstudie N‑Check geprüft.

## Studiendesign und Untersuchungsmethoden

Bei der QST handelt es sich um ein nichtinvasives psychophysisches Testverfahren. Es werden kalibrierte Reize und subjektive Empfindungsangaben genutzt, um den Funktionszustand des somatosensorischen Systems von Patient:innen zu bestimmen und zu quantifizieren [10]. Mithilfe des validierten Protokolls des Deutschen Forschungsverbands Neuropathischer Schmerz (DFNS) wird durch eine Testbatterie ein vollständiger somatosensorischer Phänotyp detektiert [11]. Diese Testbatterie besteht aus 7 Einzeltests. Insgesamt werden 13 Einzelparameter erfasst.

Die QST wurde bei den N‑Check-Patient:innen nach diesem Protokoll vor und 6–10 Wochen nach erster Chemotherapiegabe durchgeführt und in einer Prä-post-Analyse verglichen. Darüber hinaus wurden Auswirkungen der CIPN auf die Lebensqualität mittels des standardisierten und validierten Fragebogens Functional Assessment for Cancer Therapy – General (FACT-G) untersucht und objektiviert. Auch diese Ergebnisse wurden in einer Prä-post-Analyse verglichen.

## Ergebnisse

Nach positivem Ethikvotum fand zwischen Mai und November 2021 die Rekrutierung statt. Die letzte Follow-up-Untersuchung erfolgte im Februar 2022. Nach Prüfung der Ein- und Ausschlusskriterien und Absolvierung des Screenings konnten insgesamt 20 Patient:innen in die Pilotstudie eingeschlossen werden. Die erste QST konnte bei 19 Patient:innen vollständig durchgeführt werden. Das FACT‑G wurde von 18 Patient:innen beim ersten Termin vollständig ausgefüllt (Rückläuferquote 90 %).

Den zweiten Untersuchungstermin konnten 18 Patient:innen wahrnehmen. Die Rückläuferquote des FACT‑G am zweiten Untersuchungstermin betrug 88,9 % (16 von 18).

Das Patient:innenkollektiv bestand aus 10 Frauen und 8 Männern, die alle an einem soliden malignen Tumor erkrankt waren. Alle Patient:innen erhielten Platinderivate. Die verabreichten Zytostatikaregime sind Tab. 1 zu entnehmen.

**Tab. 1:** Übersicht über die Chemotherapieregime der Patient:innen

Zytostatika	Anzahl Patient:innen
Carboplatin	2
Carboplatin/Paclitaxel	2
Carboplatin/Etoposid	1
FOLFOX (Folinsäure, 5‑Fluorouracil, Oxaliplatin)	5
FOLFIRINOX (Folinsäure, 5‑Flourouracil, Irinotecan, Oxaliplatin)	3
FOLFOX/Bevacizumab	1
Oxaliplatin/Capecitabin	1
Cisplatin	2
Cisplatin/Vinorelbin	1

Neun Patient:innen gaben vor der zweiten QST neu aufgetretene neuropathische Beschwerden in den Händen oder Füßen an. Die Symptome umfassten Allodynie, Taubheit, Kribbeln und Temperatur-, insbesondere Kälteempfindlichkeit.

## QST

Die Auswertung der QST erfolgte mit den Daten der 18 Patient:innen, die beide Untersuchungen vollständig absolvierten. Bei der Analyse wurde eine Erhöhung der Hitzeschmerzschwelle (HPT) nach Beginn der Chemotherapie detektiert (Hitzehypalgesie, *p* < 0,01, siehe Tab. 2). Anderweitig zeigten sich keine signifikanten Veränderungen, auch nicht im Vergleich von Fuß und Hand der Patient:innen.

**Tab. 2:** Ergebnisse der ANOVA innerhalb des untersuchten Kollektivs

QST-Parameter	Faktor 1 Prä-post		Faktor 2 Fuß-Hand		Interaktion Faktor 1 × 2	
	*F‑Wert*	*p‑Wert*	*F‑Wert*	*p‑Wert*	*F‑Wert*	*p‑Wert*
*Kältedetektionsschwelle*	3,92	n. s.	0,45	n. s.	1,05	n. s.
*Wärmedetektionsschwelle*	0,75	n. s.	0,01	n. s.	0,04	n. s.
*Paradoxe Hitzeempfindungen*	3,76	n. s.	0,03	n. s.	0,11	n. s.
*Kälteschmerzschwelle*	1,10	n. s.	0,25	n. s.	0,62	n. s.
*Hitzeschmerzschwelle*	*12,7*	*<* *0,01*	0,87	n. s.	1,21	n. s.
*Mechanische Detektionsschwelle*	0,03	n. s.	0,04	n. s.	0,15	n. s.
*Mechanische Schmerzschwelle*	0,22	n. s.	0,01	n. s.	0,05	n. s.
*Mechanische Schmerzsensitivität*	0,39	n. s.	0,01	n. s.	0,04	n. s.
*Wind-up-Phänomen*	1,54	n. s.	0,37	n. s.	0,44	n. s.
*Vibrationsdetektionsschwelle*	0,02	n. s.	0,04	n. s.	0,03	n. s.
*Druckschmerzschwelle*	1,42	n. s.	0,29	n. s.	0,73	n. s.

*n.* *s.* nicht signifikant

## FACT-G

Für das FACT‑G ergab sich für den Prä-post-Vergleich ein Datensatz von 15 Patient:innen, die zu beiden Zeitpunkten den Fragebogen vollständig ausgefüllt hatten.

Bei den einzelnen Items zeigte sich nur bei der Aussage „Die Nebenwirkungen der Behandlung machen mir zu schaffen“ eine signifikante Erhöhung des Leidensdrucks (*p* < 0,05).

Bei den Subskalen sowie auch beim „total score“ des FACT‑G bedeutet eine höhere Zahl eine bessere Lebensqualität. Maximal können 116 Punkte erreicht werden. Von den beschriebenen Veränderungen erreichten keine das Signifikanzniveau. Es ergab sich eine Verminderung der Mittelwerte der Subskalen „personal well-being“ (PWB), „social well-being“ (SWB), „functional well-being“ (FWB) und der Subskala, welche das Verhältnis zu den behandelnden Ärzt:innen untersucht. Bei der Subskala „emotional well-being“ (EWB) ergab sich eine geringfügige Verbesserung des Scores. Daraus resultierte eine Minderung des Gesamtscores von 82,87 vor auf 76,59 nach Beginn der zytostatischen Therapie und somit eine statistisch nichtsignifikante Abnahme der Lebensqualität (Tab. 3).

**Tab. 3:** Deskriptive Auswertung des FACT‑G

FACT‑G	Mittelwert prä	Mittelwert post	Veränderung in %	*p*‑Wert
Energie	1,67	2,29	37,1	n. s.
Übelkeit	0,73	0,87	18,2	n. s.
Bedürfnisse	1,08	1,38	28,6	n. s.
Schmerzen	1,13	0,87	−23,5	n. s.
Nebenwirkungen	1,00	2,27	126,7	< 0,05
Krankheitsgefühl	1,40	1,93	38,1	n. s.
Bettlägerigkeit	0,67	1,00	50,0	n. s.
*Subskala PWB („score range“ 0–28)*	*20,18*	*17,56*	*−13,0*	*n.* *s.*
Innerliche Entfernung	0,80	0,93	16,7	n. s.
Familiäre Unterstützung	3,77	3,46	−8,2	n. s.
Soziale Unterstützung	3,33	2,53	−24,0	n. s.
Familiäre Akzeptanz	3,23	3,15	−2,4	n. s.
Kommunikation	1,75	1,75	0	n. s.
Verbundenheit	3,64	3,46	−5,0	n. s.
Sexuelle Aktivität	0,43	0,46	7,7	n. s.
Sexuelle Zufriedenheit	3,33	2,17	−35,0	n. s.
*Subskala SWB („score range“ 0–28)*	*22,80*	*20,57*	*−9,8*	*n.* *s.*
Ärztliches Vertrauen	3,40	3,27	−3,9	n. s.
Medizinische Fragen	3,53	3,27	−7,5	n. s.
*Subskala Ärzt:innen („score range“ 0–8)*	*6,93*	*6,53*	*−5,8*	*n.* *s.*
Traurigkeit	1,33	1,27	−5,0	n. s.
Bewältigung	2,60	2,40	−7,7	n. s.
Hoffnungslosigkeit	0,93	0,73	−21,4	n. s.
Nervosität	1,33	0,87	−35,0	n. s.
Angst vor dem Tod	1,33	1,27	−5,0	n. s.
Angst vor Zustandsverschlechterung	1,87	1,57	−15,8	n. s.
*Subskala EWB („score range“ 0–24)*	*15,80*	*16,75*	*6,0*	*n.* *s.*
Arbeitsfähigkeit	2,57	2,07	−19,6	n. s.
Erfüllung	2,40	2,00	−16,7	n. s.
Genuss	2,13	1,93	−9,4	n. s.
Eigene Akzeptanz	2,80	2,47	−11,9	n. s.
Schlafqualität	2,60	2,36	−9,3	n. s.
Freude	2,73	2,53	−7,3	n. s.
Lebensqualität	2,00	1,87	−6,7	n. s.
*Subskala FWB („score range“ 0–28)*	*17,16*	*15,19*	*−11,5*	*n.* *s.*
*Gesamtscore („score range“ 0–116)*	*82,87*	*76,59*	*−7,6*	*n.* *s.*

*n* = 15, *n.* *s.* nicht signifikant

## Diskussion

### Veränderungen der QST-Parameter durch die neurotoxische Chemotherapie

N‑Check als Pilotstudie hat die Einsetzbarkeit der QST zur Frühdiagnostik einer CIPN unter Einhaltung des validierten und standardisierten Protokolls untersucht.

Die Ergebnisse der QST erbrachten in der Prä-post-Analyse eine signifikante Erhöhung der Hitzeschmerzschwelle, somit eine Hitzehypalgesie, die sich mit Beginn der platinhaltigen Chemotherapie entwickelt hatte (p < 0,01). Hitzeschmerz wird von den leicht myelinisierten A‑delta-Fasern, sowie von den nichtmyelinisierten C‑Fasern übermittelt [12].

Übereinstimmende Resultate zeigte eine Studie an Patient:innen mit gastrointestinalen Karzinomen, die mit platinbasierten Therapien behandelt wurden. Bei der Untersuchung des Temperaturempfindens wiesen die Patient:innen eine erhöhte Hitzeschmerzschwelle auf [13]. In einer Untersuchung an Patient:innen, die mit Bortezomib behandelt wurden, zeigte sich ein ähnliches Ergebnis [14]. Die Testung der Hitzeschmerzschwelle ergab bereits in einer 2002 veröffentlichten Studie eine Sensitivität von 93 % für die Detektion einer Small-fiber-Neuropathie [15].

Andere Arbeiten berichten hingegen von alternativen sensorischen Abweichungen, wie z. B. Veränderungen der Vibrationsdetektionsschwelle bei Brustkrebspatientinnen, die Paclitaxel erhielten, oder mechanischen Hypästhesien und Hyperalgesien bei Patient:innen mit bekannter CIPN [16, 17].

Bei N‑Check wurden keine weiteren signifikanten Veränderungen festgestellt. Dies könnte an der früh gewählten Zweituntersuchung, dem kleinen Kollektiv im Rahmen des Studiendesigns als Pilotstudie oder auch an der Beschränkung auf platinhaltige Therapien liegen. Des Weiteren ergaben sich, im Gegensatz zu anderen Studien [10], keine Unterschiede zwischen den Werten für Fuß und Hand.

Mithilfe des Protokolls des DFNS ist es möglich, die QST-Ergebnisse unterschiedlicher Studien direkt miteinander zu vergleichen [11]. Die standardisierte Bestimmung der Hitzeschmerzschwelle könnte somit ein Parameter sein, an dem sich schon früh eine Schädigung der dünnen Schmerzfasern zeigt, und sich zukünftig wertvoll für die Diagnostik der CIPN erweisen. Allerdings lässt sich aufgrund der geringen Stichprobengröße und des frühen Untersuchungszeitpunkts hier noch keine klare Aussage bzgl. der Wertigkeit der vorliegenden Ergebnisse treffen. Weitere standardisierte Untersuchungen mit größeren Untersuchungskollektiven sind allerdings vonnöten, um diese Ergebnisse zu verifizieren.

## Die QST als verlässliche klinische Untersuchungsmethode

Die standardisierte QST ermöglicht eine genaue und verlässliche Analyse der Funktionalität des somatosensorischen Systems und erweitert die übliche klinische Elektrophysiologie um die Erfassung der Funktion dünner Nervenfasern. Zusätzlich können sensible Plus- und Minuszeichen charakterisiert werden [18]. Sie wurde entwickelt, um Limitationen der klinisch-neurologischen Untersuchung, wie z. B. die fehlende Kontrolle und Standardisierung der Stimulusintensität, zu überwinden [19]. Von der Norm abweichende QST-Werte können ein Hinweis für eine Läsion entlang der somatosensorischen Bahnen sein [20].

Die Qualifikation für die Durchführung einer QST kann durch eine Schulung erlangt werden [11]. Mit dem Untersuchungsprotokoll des DFNS wurde ein diagnostisches Instrument für einen standardisierten und damit vergleichbaren Ablauf geschaffen, welches zudem eine gute Test-Retest-Reliabilität sowie eine gute Reliabilität beim Einsatz unterschiedlicher Untersuchender bietet [21].

Weitere positive Aspekte der QST sind die Nichtinvasivität und die geringe Belastung für die Patient:innen [10]. Auch bei N‑Check kam es nicht zu unerwünschten Nebenwirkungen. Trotzdem kann die Untersuchung unangenehm sein, denn in einigen Tests werden Schmerzschwellen bestimmt. Dafür ist es nötig, diese Schwellen zu erreichen.

Für die Untersuchung von Hand und Fuß für eine QST wird etwa eine Stunde benötigt [11]. Diese Zeitspanne wurde von allen Patient:innen, mit Ausnahme einer Patientin, problemlos akzeptiert.

Die QST als Methode erfordert die Mitarbeit der untersuchten Personen. Die sensorischen Stimuli sind objektive physikalische Ereignisse, die Reaktionen beruhen jedoch auf subjektiven Empfindungen [22]. Im Protokoll des DFNS werden die Tests daher standardisiert erklärt. Somit ist es allerdings unerlässlich, dass die untersuchten Personen kognitiv in der Lage sind, den Anweisungen Folge zu leisten. Sprachbarrieren, eine verminderte Aufmerksamkeit oder andere Einschränkungen können die Ergebnisse verfälschen.

Die QST ist in vielen klinischen Bereichen bereits ein etabliertes diagnostisches Instrument. So findet sie z. B. Verwendung bei der Diagnostik der diabetischen Polyneuropathie, der Fibromyalgie und Small-fiber-Neuropathien unterschiedlicher Genese [15, 23, 24]. Martland et al. beleuchten in ihrer systematischen Übersichtsarbeit den Einsatz der QST bei der Beurteilung von Krebsschmerzen. In 15 Studien zeigten sich sensorische Auffälligkeiten in der QST aufgrund einer CIPN [25].

Ein Goldstandard ist bisher in der Diagnostik der CIPN nicht etabliert. Die Kombination verschiedener Untersuchungen, wie der klinisch-neurologischen Untersuchung, der QST, der Nervenleitgeschwindigkeitsmessung oder der evozierten Potenziale, scheint momentan die erfolgversprechendste Herangehensweise zu sein [26]. Allerdings könnte die Durchführung von vier verschiedenen Untersuchungen eine hohe Belastung für die Patient:innen bedeuten. Unsere Pilotstudie fokussierte sich u. a. aus diesem Grund auf die Ergebnisse der QST. Ob das standardisierte Vorgehen der QST dabei ausreichend ist, um eine Frühdiagnostik der CIPN zu erreichen, und die Hitzehypalgesie ein richtungsweisender diagnostischer Wert ist, müssen folgende Studien mit größeren Kollektiven prüfen.

## Evaluation der gesundheitsbezogenen Lebensqualität

Um die krebsspezifische gesundheitsbezogene Lebensqualität zu untersuchen, wurde die dritte Version des FACT‑G genutzt. Grund für die Wahl dieser Version war die Möglichkeit der Bewertung des Verhältnisses zu den behandelnden Ärzt:innen [27].

Das Gesamtergebnis zeigte eine nichtsignifikante Verschlechterung der Lebensqualität.

Lediglich das Item, das den Leidensdruck unter den Nebenwirkungen der Therapie beleuchtet, zeigt eine signifikante Veränderung. Das FACT‑G lässt eine genauere Aufschlüsselung über die spezifische Symptomlast nicht zu. Dennoch könnte eine CIPN die Ursache für diesen erhöhten Leidensdruck sein, welcher die globale Lebensqualität allerdings nicht signifikant verschlechterte.

Mols et al. konnten in ihrer Übersichtsarbeit eine deutliche negative Assoziation zwischen CIPN und Lebensqualität herausstellen. Eines der in den analysierten Studien eingesetzten Instrumente war das FACT‑G [28]. Hung et al. untersuchten das Auftreten einer platininduzierten Polyneuropathie bei Lungenkarzinompatient:innen und den Einfluss dieser auf die Lebensqualität mittels FACT‑G. Eine niedrige Lebensqualität war assoziiert mit vermehrten CIPN-Symptomen und einem höheren Grad an Depressionen und Angstzuständen [29].

Bei einem größeren Untersuchungskollektiv könnte der Einsatz des FACT‑G somit dennoch eine signifikante Abnahme der Lebensqualität, die mit dem Vorhandensein einer CIPN eintreten kann, hervorbringen. Ein weiterer Grund, warum die Minderung der Scores kein signifikantes Niveau erreichte, ist die frühzeitige Zweitevaluation. Bei einigen Patient:innen vergingen nur sechs Wochen zwischen den Terminen, sodass eine signifikante Abnahme der Lebensqualität mit einer dramatischen Änderung der Lebenssituation einhergehen hätte müssen.

## Limitationen

N‑Check zeigt einen vielversprechenden Ansatz zur Frühdiagnostik der CIPN. Dennoch ist insbesondere die geringe Fallzahl, die die Übertragbarkeit der erhobenen Daten einschränkt, als Limitation einzuschätzen. Des Weiteren wurden zwar nur Patient:innen in die Studie eingeschlossen, bei denen ein solider Tumor mit Platinderivaten behandelt wurde, doch gab es keine genauere Eingrenzung der Tumorentität oder des Metastasierungsstatus. Andere antitumoröse Vorbehandlungen oder Vorerkrankungen wurden im Screening nicht berücksichtigt, da der Fokus auf dem Ausschluss einer klinisch detektierbaren Neuropathie vor Einschluss in die Pilotstudie lag. Die Eingrenzung der verabreichten Chemotherapien umfasste nur Platinderivate, eine Spezifizierung auf ein bestimmtes Platinderivat oder bestimmte Kombinationstherapien mit anderen Präparaten fand nicht statt. Die kurze Nachbeobachtungszeit, die sich aus der Fokussierung auf die Frühdiagnostik der CIPN im Rahmen dieses Pilotprojekts ergibt, schränkt zudem die Erfassung von Langzeiteffekten der Chemotherapie auf die Nervenfunktion und die Lebensqualität ein. Berücksichtigt werden sollte außerdem, dass sowohl die QST als auch das FACT‑G auf subjektiven Wahrnehmungen der Patient:innen beruhen. Die Kombination mit objektiven Untersuchungsmethoden in Folgestudien sollte die Validität von QST und FACT‑G in diesem Zusammenhang überprüfen.

Künftige Studien könnten unter Berücksichtigung dieser Punkte noch aussagekräftigere Ergebnisse erhalten. Insbesondere die Vergrößerung der untersuchten Gruppe ist notwendig, um die Reliabilität der von uns erhobenen Daten zu testen.

## Fazit für die Praxis

- Die standardisierte Durchführung der QST vor und nach Beginn neurotoxischer Chemotherapie konnte eine Grundlage für die CIPN-Frühdiagnostik schaffen. Die Patient:innen zeigten an T2 eine Hitzehypalgesie, somit Anzeichen einer Schädigung kleiner Nervenfasern. Die QST erwies sich als unkomplizierte und wenig belastende Untersuchung.
- Die FACT-G-Auswertung deutete die Abnahme der Lebensqualität unter potenziell neurotoxischer Zytostatikatherapie an. Trotz neuer Therapieansätze werden konventionelle Zytostatika vorerst Teil von Tumortherapien, v. a. auch im palliativen Kontext, bleiben. Gerade hier sollten Nebenwirkungen vermieden werden, sodass N‑Check und die beantragte Folgestudie einen Beitrag zur Lebensqualitätssteigerung dieser Patient:innen leisten.

## Data Availability

Die Rohdaten zur vorliegenden Studie sind über die Autor:innen erreichbar.
